# The effect of Japanese eel as a main ingredient on hair condition, antioxidant ability, apparent total tract digestibility and body weight gain in cat food

**DOI:** 10.3389/fvets.2025.1553320

**Published:** 2025-06-16

**Authors:** Yilin Yuan, Leilei Zhu, Xuan Cai, Hui Mao, Tingting Wang, Shengqing Tao, Jiayu Bao, Chengyin Liu, Jie Feng

**Affiliations:** ^1^Key Laboratory of Animal Nutrition and Feed Sciences of Zhejiang Province, College of Animal Science, Zhejiang University, Hangzhou, China; ^2^Hangzhou PETISALL Biotechnology Co., Ltd., Hangzhou, China; ^3^Institute of Animal Science and Veterinary Medicine, Shanghai Academy of Agricultural Science, Shanghai, China; ^4^PETIDEAL Pet Food (Luohe) Co., Ltd., Luohe, China

**Keywords:** Japanese eel, cat food, polyunsaturated fatty acids, hair condition, antioxidant

## Abstract

This study aims to evaluate the impact of Japanese eel (*Anguilla japonica*) as a primary ingredient in cat food on hair condition, antioxidant capacity, apparent total tract digestibility (ATTD), and body weight gain in adult cats. Twenty-four healthy adult cats were assigned divided into three dietary groups: a 0% eel group (C), a 14% eel group (T1), and a 40% eel group (T2). Over a 56-day period, hair coat condition, ATTD, antioxidant indices, and body weight were monitored. The results indicated an improvement in hair luster and softness among the high eel group. By day 56, the 40% eel group demonstrated significant improvements in both hair luster (3.0 vs. 2.4 in control, *p* < 0.05) and softness (3.0 vs. 2.2 in control, *p* < 0.05). Scanning Electron Microscopy (SEM) analysis confirmed a significant reduction in hair scale thickness for the 14% (39.36% reduction) and 40% eel groups (46.80% reduction) compared to the control group (*p* < 0.05). The ATTD of calcium was significantly higher in both eel groups (71.3% for T1 and 74.1% for T2 vs. 62.8% in control, *p* < 0.05); however, no significant differences were observed regarding crude fat or protein digestibility (*p* > 0.05). Serum antioxidant capacity was notably enhanced in the 14% eel group, with a 23% increase in total antioxidant capacity (T-AOC) and a 15% increase in superoxide dismutase (SOD) activity compared to the control (*p* < 0.05). Furthermore, glutathione (GSH) content was higher, and malondialdehyde (MDA) levels were lower in the eel groups; however, these differences did not reach statistical significance (*p* > 0.05). Notably, body weight gain showed a slight downward trend in the eel groups, with average daily gain recorded at 2.1 g/day for T2 versus 2.4 g/day for the control group, though this difference was not significant (*p* > 0.05). Fecal scores remained consistent across all experimental groups, indicating no adverse effects on fecal quality (*p* > 0.05). These findings suggest that incorporating Japanese eel into cat food can enhance hair coat condition and antioxidant capacity without adverse effects on body weight or fecal quality. Therefore, it is recommended to include a 14% level for optimal benefits.

## 1 Introduction

Global production of aquatic animals was estimated at 178 million tons in 2020 (FAO, 2022), while global pet food production is projected to reach 35.27 million tons in 2022, according to a report by Alltech. Among pets, the significance of cats cannot be overlooked. Accurately determining the exact number of cats worldwide poses challenges due to varying estimates from different organizations, resulting in substantial discrepancies in data. A 2020 report indicated that there were at least 220 million pet cats and 480 million stray cats worldwide (1). The high-protein nature of marine food renders it particularly suitable as an ingredient for cats, which are primarily carnivorous. However, research on seafood as a primary ingredient in cat food remains limited.

As public awareness regarding pet nutrition deepens, there is a growing recognition that high-quality protein and fat sources are essential for maintaining pet health (2, 3). At the same time, the global population of pets is rising rapidly, leading to a surge in demand for premium pet foods and raw materials (4). Therefore, it is crucial to acknowledge that our land resources are finite, and priority must be given to providing food for humans.

The Japanese eel belongs to the Anguilla genus and is commonly cultivated and consumed as food across East Asian countries such as China, Japan, and South Korea (5). In recent years, China’s annual eel output has surpassed 2.2 million tons, accounting for approximately 70% of the world’s total output (6). The majority of these eels are processed prior to sale, through various techniques including smoking, jellying, pickling, and kabayaki—a method specifically tailored for the Japanese market.

The study conducted by Seo et al. (7) revealed eel meat contains approximately 16–18% protein, and 10–20% lipid; notably rich in highly unsaturated (n-3) fatty acids with content levels ranging from 25 to 33%. Eicosapentaenoic acid (EPA) and docosahexaenoic acid (DHA) comprise up to 24–32% of the total fatty acids (7). This implies that eels may serve as valuable raw materials for pet food; however, there is currently a lack of reports regarding their health effects. Previous data have shown that diets rich in n-3 fatty acids can enhance various aspects of pet health, including coat quality (8, 9), antioxidant capacity (10), lipid metabolism (3), and immune function modulation (9).

Although extrusion technology remains the predominant method for processing dry pet food, a significant portion is produced through baking methods (11). Baked foods exhibit lower levels of rancidity indicators, which can enhance palatability for pets (12). More importantly, numerous studies have demonstrated that thermal processing affects the stability of polyunsaturated fatty acids (13, 14). Therefore, all diets in this study were prepared using low-temperature baking processing techniques.

To further explore the potential application of eel in pet food, this study substitutes chicken with Japanese eel as the primary source of protein and fat in cat food. It systematically evaluates the effects of eel-based diets on hair coat condition, antioxidant capacity, apparent total tract digestibility (ATTD), and changes in body weight among adult cats. The study aims to assess both the feasibility and benefits associated with incorporating eel as a nutritional ingredient to enhance feline health. This will provide scientific support for developing cat food products containing eel-based ingredients.

## 2 Materials and methods

### 2.1 Animals and experimental design

Twenty-four healthy adult cats with body weight 3.61 ± 0.69 kg were used in this study. The experiment was conducted at the Shanghai Academy of Agricultural Sciences. Prior to the commencing the study, the health status of each cat was confirmed by veterinarians from Shanghai Academy of Agricultural Sciences Pet Hospital.

From 3:00 p.m. to 9:00 a.m. (18 consecutive hours), each cat was housed individually in its separate cage (80 × 70 × 60 cm), equipped with a feeding bowl, a drinking bowl, and a litter box. The experimental site was maintained at a temperature of 22 ± 3°C and a humidity level of 60 ± 5%, with artificial lighting, a humidifier and an air conditioner. Throughout this period, each cat had access to adequate water and food. From 9:00 a.m. to 3:00 p.m. (six consecutive hours), the cats were housed in a collective cattery (20 m^2^ and 4 m high), with multi-layered climbing frames for exercise and socialization purposes while allowing access to sunlight. During this entire period, the cattery was equipped with drinking bowls and litter boxes; however, they had access to water but no food.

After a one-week adaptation period, the cats were randomly assigned into three groups (C: 0% eels, T1: 14% eels, T2: 40% eels), with each group consisting of eight independent replicates. Each group maintained consistent breed compositions, including 2 British Shorthairs, 3 Domestic Shorthairs, 1 American Shorthair, and 2 Ragdolls (The detailed information on experimental animals was listed in Supplementary material 1).

During the experiment’s duration of 56 days, all cats had free access to both food and water. On days 0, 28 and 56, body weight measurements for each cat were taken. Food intake was measured daily.

Feces were subjectively scored daily according to the method described by “The Waltham® Faeces Scoring System,” where scores ranged from 1 = hard dry and crumbly; 2 = well-formed without leaving marks when picked up; 3 = moist at the beginning and then losing its form while leaving a definite mark when picked up; 4 = the majority, if not all forms are lost; 5 = watery diarrhea.

The method described by Guo et al. (15) was adopted for assessing hair scores on day 0, 28 and day 56. In brief, the hair condition of cats at the conclusion of each stage was assessed using a subjective scoring method conducted by 5 professional breeders blinded to the study. All samples received scores based on 3 increments using the following scale:

Hair brightness: 1 = dull, 2 = medium reflective, 3 = bright.

Hair softness: 1 = coarse, 2 = medium soft, 3 = very soft.

### 2.2 Diets

Three diets with different eel content (0, 14, 40%) were formulated by Hangzhou PETISALL Biotechnology Co., Ltd. to meet all Association of American Feed Control Officials (AAFCO) (16) nutrient profiles for adult cats at maintenance. The eels used in the experiment weighed approximately 250 g each, and were immediately stored at −60°C after being caught, and the whole eels served as raw material for processing. The other ingredients, excluding eels and chicken, remained consistent across the three diets. Detailed information regarding the diets can be found in Table 1.

**Table 1:** Ingredient composition of eel-containing experimental diets^1^.

Ingredients	C, 0% eels	T1, 14% eels	T2, 40% eels
Frozen chicken breast	68	54	28
Japanese eel	0	14	40
Frozen duck	12	12	12
Fresh chicken liver	8	8	8
Frozen chicken heart	4	4	4
Fresh sweet potato	2.4	2.4	2.4
Cassava starch	1.3	1.3	1.3
Vitamins and minerals premix^2^	1.2	1.2	1.2
Fish oil	1	1	1
Cellulose	1	1	1
Yolk power	0.6	0.6	0.6
Dry apple power	0.2	0.2	0.2
Cheese powder	0.2	0.2	0.2
Cranberry powder	0.1	0.1	0.1

1 as feed, %.

2 Provided per kilogram of diet: vitamin A, 22,000.00 IU; vitamin D, 3,500.00 IU; vitamin E, 50.00 mg; vitamin K, 0.10 mg; vitamin B_1_, 18.00 mg; vitamin B_2_,7.50 mg; vitamin B_3_, 95.00 mg; vitamin B_6_, 8.50 mg; vitamin B_12_, 0.03 mg; calcium pantothenate, 9.50 mg; D-biotin, 0.11 mg; folic acid, 0.90 mg; cholinechloride, 2600.00 mg; Fe, 80.00 mg; Cu, 15.00 mg; Mn, 7.80 mg; Zn, 75.00 mg; I, 1.88 mg; and Se, 0.30 mg.

The dry matter, crude protein, fat (ether extract), total Ca and P contents of the diet were determined according to the description by Association of Official Analytical Chemists (35), respectively.

The content of fatty acid in feed was assessed by an internal standard method, conforming to Chinese national standards (17).

### 2.3 Sample collection

From day 50 to day 56, cat litter was removed from the boxes and incontinence pads were stuck to the boxes to facilitate feces collection. These pads absorb urine, thereby making feces easier to collect. Most cats tend to refuse defecation during the first 2 d of using incontinence pads but gradually adapt after 3 d. The incontinence pads were replaced daily. And feces from each cat were collected from their respective litter boxes per day. Feces contaminated with hair, incontinence pads or other foreign matter were discarded. All feces from one cat were composited with 10 mL of 5% sulfuric acid, and frozen at −20°C until analyses. The crude protein, fat (ether extract), total Ca and P of feces were determined according to Association of Official Analytical Chemists (35) methods. Acid-insoluble ash served as an indigestible marker (18) and was analyzed using the method described by Cai et al. (19). The apparent total abstract digestibility of nutritional components was calculated by the following formula:


$$\begin{array}{l} \text{Apparent digestibility of}\;a\;\text{nutrient}=\\ (1-\frac{\mathrm{AI}A_{\text{feed}}\times \text{Nutrien}t_{\text{feces}}}{\mathrm{AI}A_{\text{feces}}\times \text{Nutrien}t_{\text{feed}}})\times 100\% \end{array}$$

Among them, $\mathrm{AI}A_{\text{feed}}$ is the content of acid-insoluble ash in feed, $\text{Nutrien}t_{\text{feces}}$ is the content of a specific nutrient within feces.

The hair on back of all cats’ necks was shaved on day 0, and samples of hairs for Scanning Electron Microscopy (SEM) were collected on day 28 and 56 by a hair clipper at the back of their necks symmetrically (hair collected on day 28 from left side, on day 56 from right side).

Blood samples for 3-5 mL were collected from each cat via the forelimb vein on day 56, blood was transferred to a 15 mL serum separator tube and left to stand for 60 min at room temperature before centrifugation at 3,500 g, 4°C for 15 min. After centrifugation, the supernatants were aliquoted into microcentrifuge tubes and stored at −20°C for further analyses.

### 2.4 Scanning electron microscopy

The histological evaluation was performed by using SEM (Ri Li SU8010, HITACHI, Japan). The sample was pretreated by the method of Guo et al. (15). Image evaluation was performed blindly by independent observers. On the basis of the SEM images, the arrangement of scales in relation to the longitudinal axis of hair, the type of cuticle, the structure of edges of the cuticles, and the scale height were determined.

### 2.5 Apparent digestibility of nutrients

The pelleted diets were ground to pass through a 0.5-mm screen. Feces were dried in an air-dry oven at 60°C for 48 h, and then ground to pass through a 0.5-mm screen. The dry mass, crude protein, fat (ether extract), total Ca and P contents of the excreta and diet were determined according to the description by Association of Official Analytical Chemists (35). Acid-insoluble ash was used as an indigestible marker and analyzed using the method of Silva et al. (20).

### 2.6 Serum biochemical analysis

For antioxidant index analysis, samples were analyzed according to the manufacturer’s instructions, the antioxidant ability was evaluated by determining the activities of Superoxide dismutase (SOD, product number: A001-3-2, Nanjing Jiancheng Bioengineering Institute, Jiangsu, China, NBI) via water-soluble tetrazolium salt-1 (WST-1) method, and glutathione (GSH, product number: A006-2-1, NBI) via 5,5′-dithiobis (2-nitrobenzoic acid) (DTNB) method, as well as total antioxidant capacity (T-AOC, product number: A015-2-1, NBI) via 2,2′-Azino-bis (3-ethylbenzothiazoline-6-sulfonic acid) (ABTS) method, and malondialdehyde (MDA, product number: A003-1-2, NBI) content via thiobarbituric Acid (TBA method).

For other analysis, samples from each group were randomly mixed into 3 aliquots. The concentrations of serum total protein were analyzed using bicinchonininc acid method; serum albumin was assessed by bromcresol green method; serum triglyceride were analyzed by glycerol-3-phosphate oxidase / perioxidase (GPO-PAP) method; serum total cholesterol (TC) were analyzed by cholesterol oxidase/ perioxidase (COD-PAP) method; alanine aminotransferase (ALT) activity was analyzed by Reitman Frankel method. All biochemical indices in serum were measured using commercial assay kits from Nanjing Jiancheng Bioengineering Institute, with product numbers A045-4-1 for total protein, A028-2-1 for albumin, A110-1-1 for triglyceride, A111-1-1 for cholesterol, and C009-2-1 for ALT.

### 2.7 Statistical analyses

All data are presented as mean ± standard deviation (SD). Means were compared using ANOVA with post-hoc testing (Duncan’s Multiple Range Test). Differences between treatment means were significant at *p* < 0.05 with trends identified when *p* ≥ 0.05 but <0.10. All statistical analyses were performed using SPSS 26.0 software and plotted with the Graphpad Prism 8.0 software.

## 3 Results

### 3.1 Chemical composition of feed

Eel-containing diets showed a slight reduction in crude protein and crude fat (ether extract) compared to chicken-only diet (Table 2). Meanwhile, there was an increase in the levels of crude fiber, crude ash, calcium, and total phosphorus. Fatty acid analysis indicated an increase in both n-3 fatty acids (including cis-4,7,10,13,16,19-docosahexaenoic acid, cis-5,8,11,14 17-eicosapentaenoic acid and cis-9 12 15-octadecatrienoic acid) and n-6 fatty acids (including cis-8 11 14-eicosatrienoic acid and cis-9 12-octadecadienoic acid) in the eel-containing diet compared to the pure chicken raw material diet. The total polyunsaturated fatty acids in 14 and 40% eel staple food are 58 and 82%, respectively.

**Table 2:** The chemical and energy compositions of eel-containing experimental diets.^1^

Parameter	C, 0% eels	T1, 14% eels	T2, 40% eels
Crude protein, CP	44.70	44.05	43.58
Ether extract, EE	19.73	18.91	18.12
Crude fiber, CF	1.54	2.50	3.70
Ash	7.37	8.00	8.10
Calcium, Ca	1.29	1.35	1.54
Total phophorus, TP	1.01	0.91	1.25
Gross energy (kJ/g)	23.26	22.92	22.70
Fatty acid			
C24:1n9 cis-15-tetradecanoic acid	0.00	0.00	0.40
C22:6n3 cis-4,7,10,13,16,19-docosahexaenoic acid	0.09	0.33	0.43
C21:0 Heneicosanoic acid	0.00	0.00	0.06
C22:1n9 cis-13-docosahexaenoic acid (erucic acid)	0.30	0.34	0.00
C20:5n3 cis-5,8,11,14,17-eicosapentaenoic acid	0.08	0.27	0.24
C20:3n6 cis-8,11,14-eicosatrienoic acid	0.06	0.07	0.06
C20:1 cis-11-eicosenoic acid (arachidonoic acid)	0.07	0.08	0.10
C18:3n3 cis-9,12,15-octadecatrienoic acid (α-linolenic acid)	0.18	0.28	0.31
C18:2n6c cis-9,12-octadecadienoic acid (linoleic acid)	2.46	3.58	4.20
C18:1n9c cis-9-octadecenoic acid (oleic acid)	4.36	5.20	5.52
C18:0 Octadecanoic acid (stearic acid)	1.15	1.26	1.21
C17:0 Heptadecanoic acid	0.00	0.05	0.00
C16:1n7 cis-9-hexadecenoic acid (palmitoleic acid)	0.64	0.80	0.59
C16:0 Hexadecanoic acid (palmitic acid)	3.74	3.87	3.34
C14:0 Tetradecanoic acid (myristic acid)	0.14	0.24	0.25
n-3 fatty acid	0.36	0.88	0.98
n-6 fatty acid	2.52	3.65	4.26
Polyunsaturated fatty acids	2.88	4.53	5.24

1 dry basis, %.

### 3.2 Food intake and body weight

**Figure 1:**
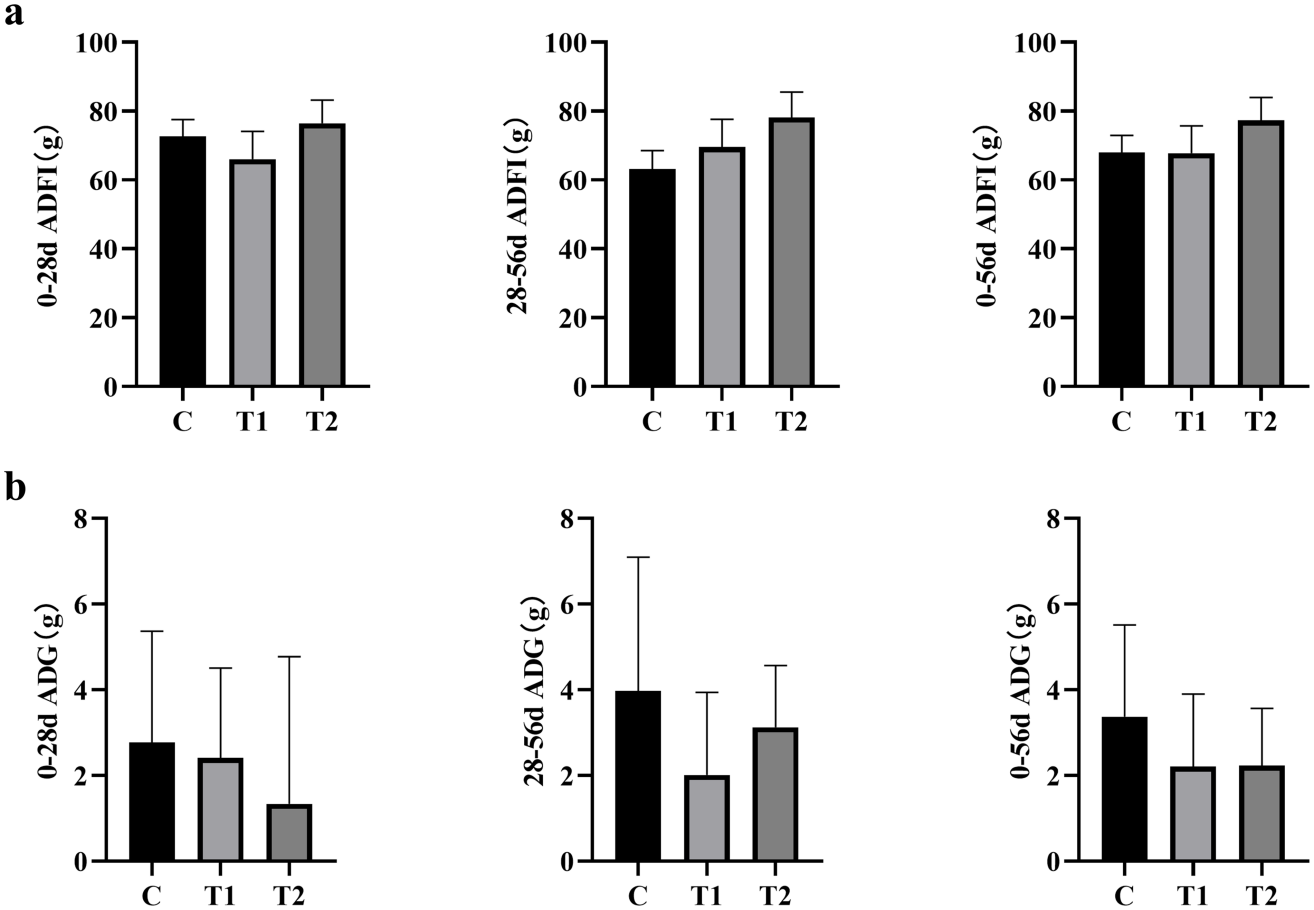
Effect of eel supplementation on cat **(a)** feed intake and **(b)** weight gain. ADFI, average daily feed intake; ADG, average daily weight gain; *n* = 8.

The average daily food intake is shown in Figure 1a. Throughout all experimental phases, there were no significant differences in average daily food intake among the groups of cats. (*p* > 0.05). Additionally, as depicted in Figure 1b, there was also no statistically significant difference observed in the average daily weight gain of the cats (*p* > 0.05).

### 3.3 Apparent total tract digestibility (ATTD)

**Figure 2:**
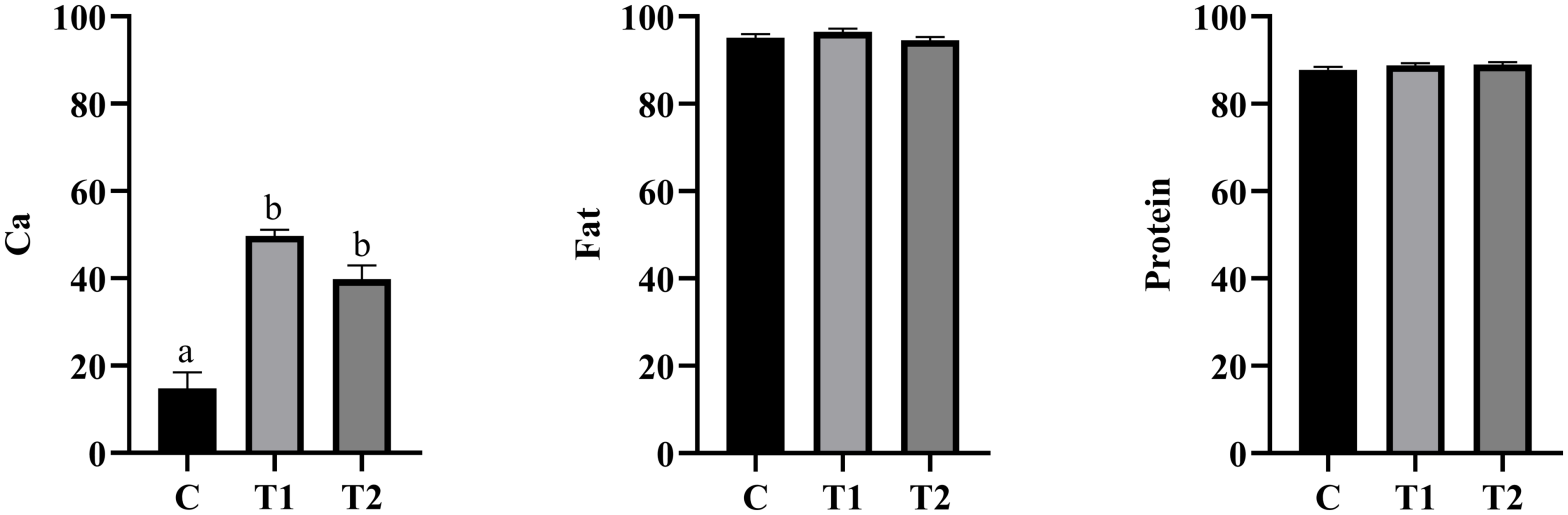
Apparent total tract digestibility. a,b, Different letters indicate significant differences among components, the same applies below; *n* = 8.

As shown in Figure 2, the apparent digestibility of Ca in the experimental group was significantly increased (*p* < 0.05) compared to the control group. Among them, the apparent digestibility of Ca in the experimental group that consumed 14% eel staple food was found to be the highest. However, compared to the control group, there was no significant difference in the apparent digestibility of both crude fat and crude protein between the experimental group that consumed eel staple food and the control group (*p* > 0.05).

### 3.4 The impact of hair quality

**Figure 3:**
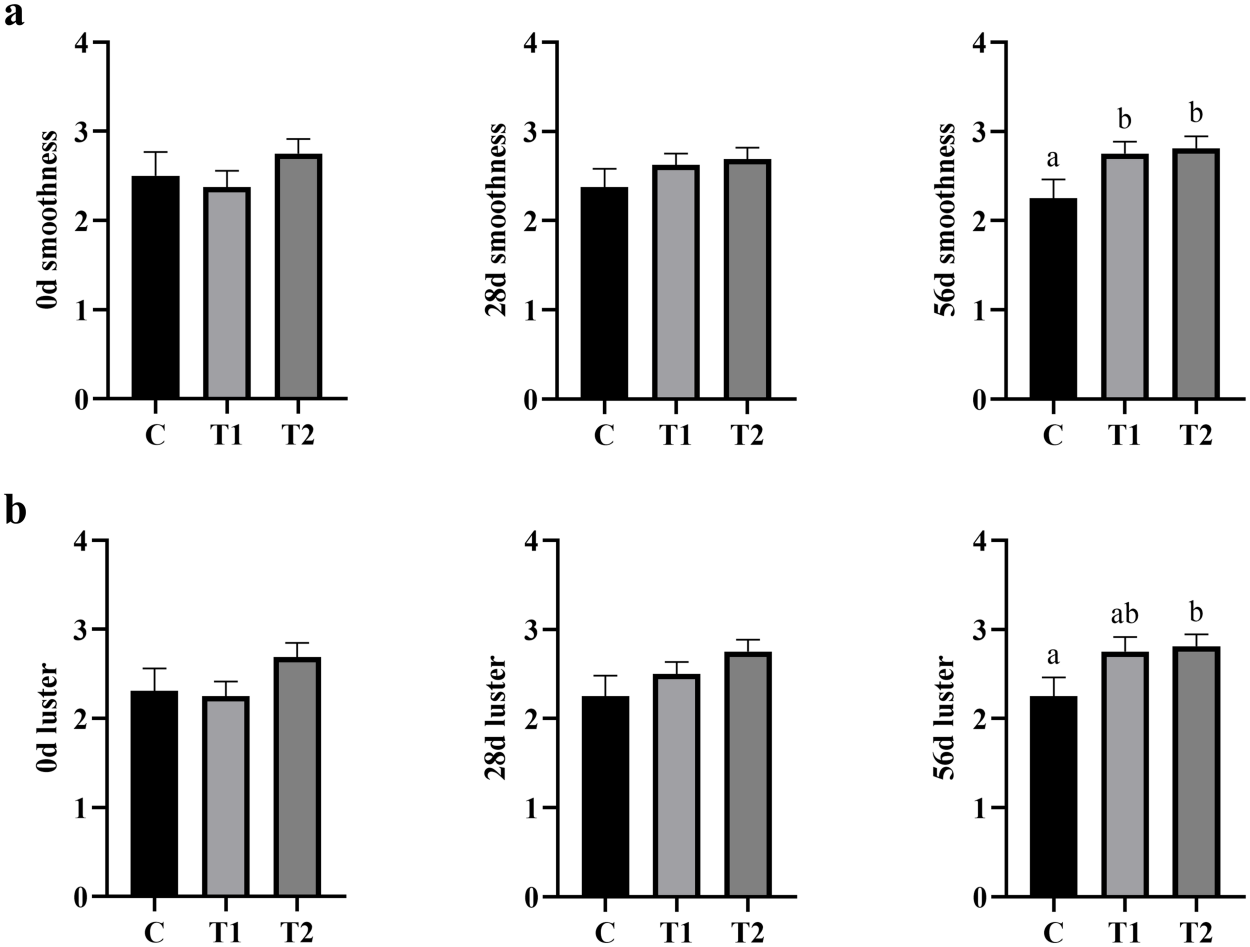
Effect of eel supplementation on hair smoothness and luster. **(a)** Hair smoothness; **(b)** hair luster.

The effect of the eel-containing diet on hair characteristics is presented in Figure 3. Initially, there were no significant differences in hair smoothness (Figure 3a) among the three groups at day 0 (*p* > 0.05). After 28 days, there was no statistically significant difference in hair smoothness between the experimental and control groups, although an increasing trend (*p* = 0.065) was observed with higher eel inclusion. By day56, hair smoothness in the 40% eel group was significantly higher than that in the control group (*p* < 0.05). Similarly, no initial differences in hair luster (Figure 3b) were found among the groups at day 0 (*p* > 0.05). After 28 days, hair luster remained statistically similar between the experimental and control groups (*p* > 0.05). However, at 56 days hair luster in the eel-containing group was significantly greater than that of the control group (*p* < 0.05).

**Figure 4:**
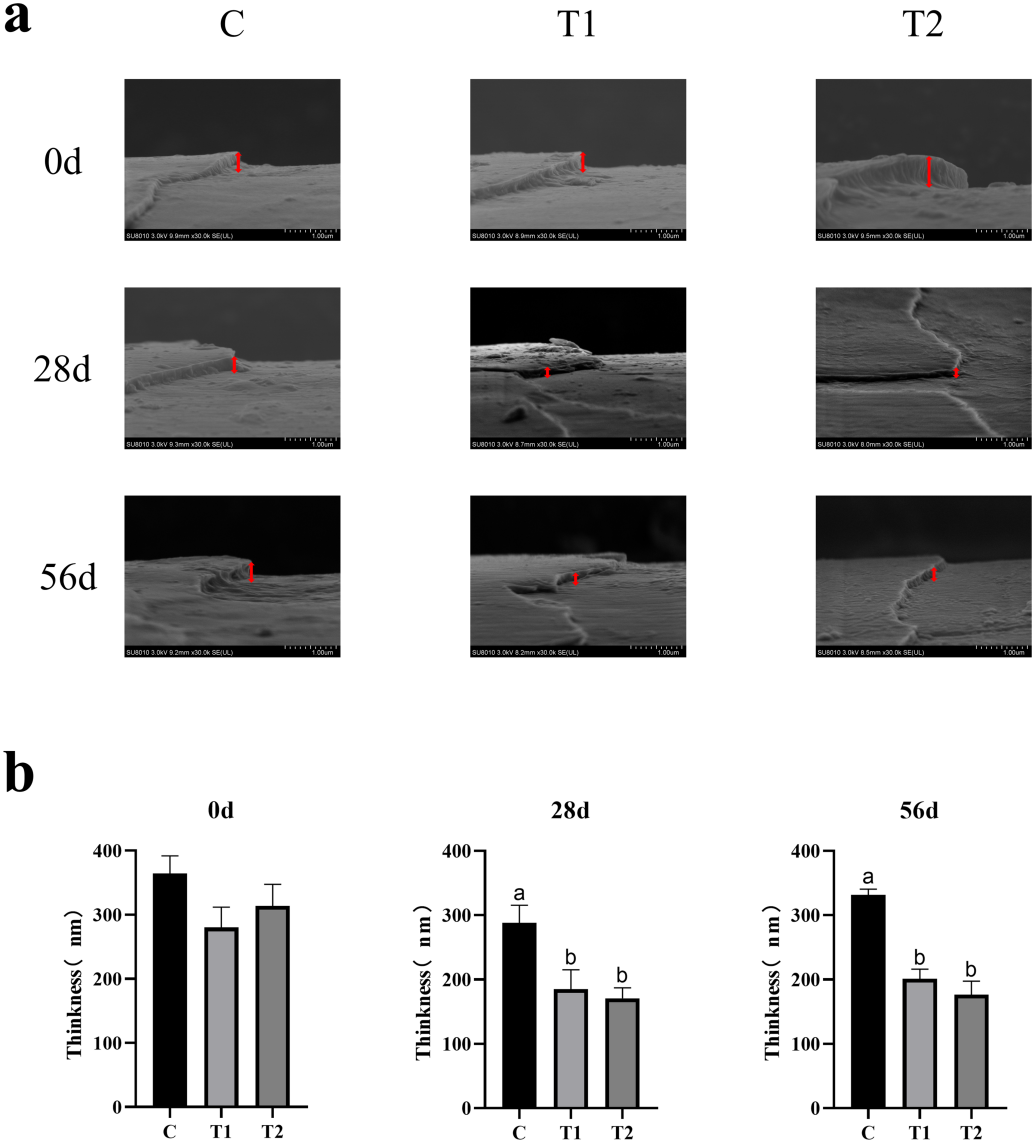
Effect of eel supplementation on hair quality: **(a)** Hair scale thickness; **(b)** The impact of consuming eel containing feed on hair scale thickness.

Scanning Electron Microscopy (SEM) was used to investigate changes in hair quality (Figure 4a). After 28 and 56 days of consuming an eel-containing diet, there was a significant decrease in hair scale thickness (*p* < 0.05). At day 28, the hair scale thickness in the 14% eel group was reduced by 35.75% compared to the control group, and by 40.71% in the 40% eel group (Figure 4b). At day 56, the 14% eel group showed a 39.36% reduction, and the 40% eel group showed a 46.80% reduction in hair scale thickness compared to the control group (Figure 4b).

### 3.5 The influence on subjective fecal score

**Figure 5:**
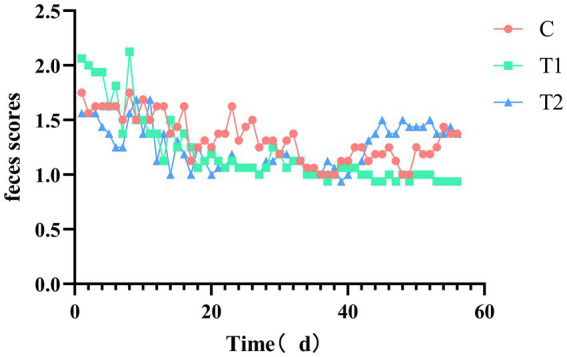
Effect of eel supplementation on feces scores.

The impact of adding eel staple food on cat feces scores is shown in Figure 5. The feces scores of the experimental group fed with food containing 14% eel showed a downward trend from 1 d to 28 d, and then stabilized in all the cats. The fecal score of the experimental group fed with 40% eel food showed not changed from 1 d to 28 d, but an upward trend from 28 d to 56 d. The feces score of the group that consumed eel-containing staple food reached its highest around 28 days.

### 3.6 Serum antioxidant index

**Figure 6:**
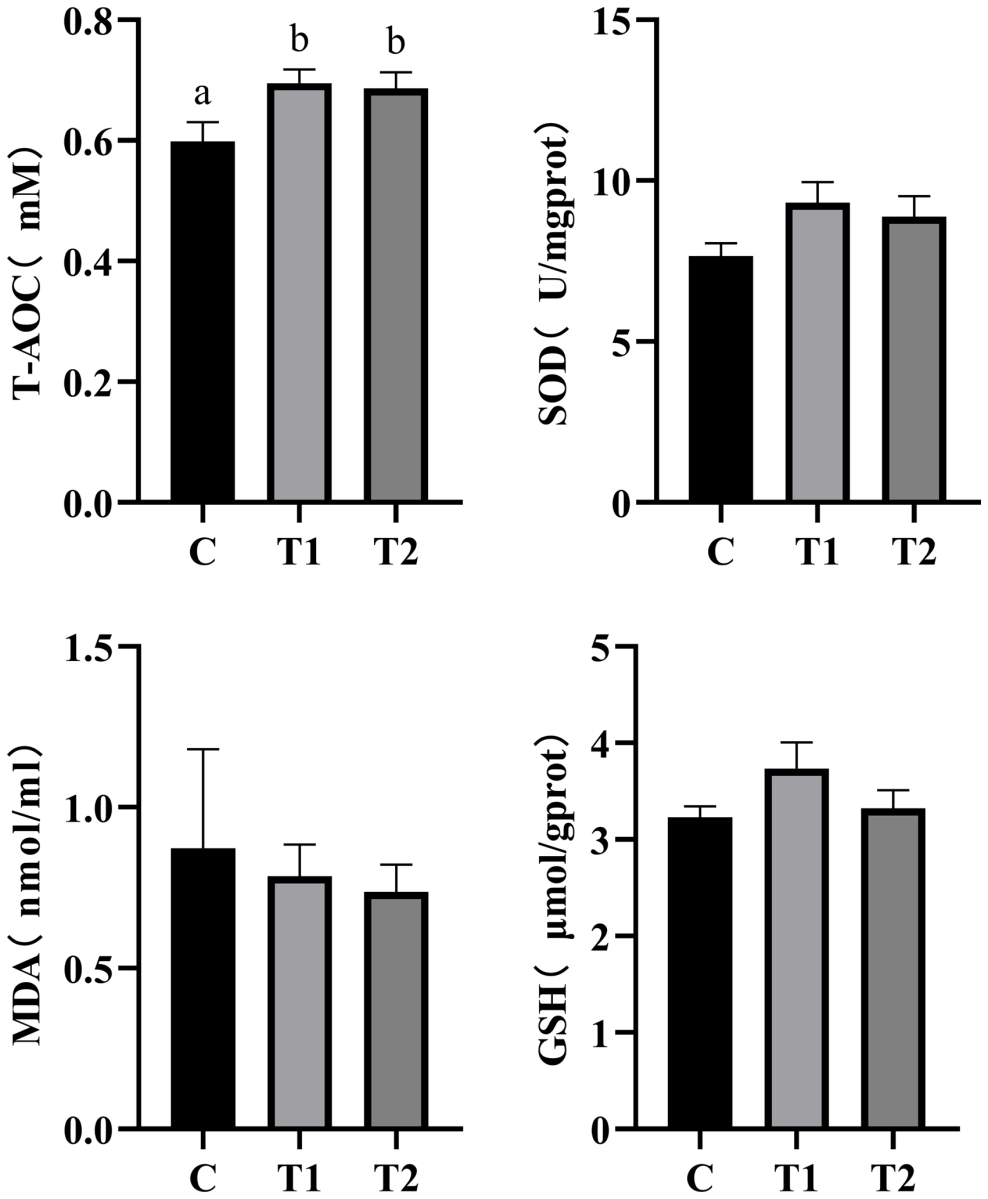
Effect of eel supplementation on antioxidant capacity. SOD, superoxide dismutase; MDA, malondialdehyde; GSH, glutathione. Different letters (a and b) in the figure indicate significant differences (*P* < 0.05) between groups, *n* = 8.

Free radical metabolism is associated with various factors such as immunity and aging. SOD, MDA, and GSH are crucial antioxidant indicators whose activity/concentration reflects the body’s antioxidant capacity. As shown in Figure 6, at the end of this experiment (56 d), compared to the control group, the eel-containing groups (T1, T2) exhibited a significant increase in total antioxidant capacity in serum (*p* < 0.05). The cats fed a diet containing 14% eel (T1) showed the most pronounced improvement in antioxidant capacity. Compared to the control group, cats fed eel-containing diets did not showed statistically differences in serum SOD activity, GSH content, and MDA content (*p* > 0.05).

### 3.7 Other serum biochemical indexes

Table 3 shows that there were no significant differences (*p* > 0.05) among cats fed different diets regarding serum total protein (TP), albumin (ALB), total cholesterol (TC), or alanine aminotransferase activity (ALT) among cats fed different diets. It was noted that serum triglyceride (TRIG) in cats fed diets containing eel meal (T1 and T2 groups) were significantly lower than those observed in the control group (*p* < 0.05). However, no significant difference (*p* > 0.05) was observed ibetween T1 and T2 groups concerning their respective serum triglyceride levels.

**Table 3:** The serum biochemical index of cats feed with different diets^1^.

Items	C, 0% eels	T1, 14% eels	T2, 40% eels
Total protein, g/L (TP)	62.807 ± 3.006	60.991 ± 2.837	62.480 ± 1.800
Albumin, g/L (ALB)	38.565 ± 2.707	39.947 ± 1.913	40.556 ± 1.538
Triglyceride, mmol/L (TRIG)	0.454 ± 0.028^a^	0.345 ± 0.021^b^	0.321 ± 0.019^b^
Total cholesterol, mmol/L (TC)	2.092 ± 0.125	1.994 ± 0.171	1.919 ± 0.096
Alanine aminotransferase, U/L (ALT)	28.883 ± 3.081	29.544 ± 1.565	28.088 ± 3.556

1 Different superscript letters in the same row indicate significant differences (*p* < 0.05), *n* = 3.

## 4 Discussion

According to report of World Integrated Trade Solution (36), China is the second largest exporter of eel in the world, with a total export volume of 252 million US dollars in 2022. However, there is still little research on the application of eel in pet food.

This study reveals that pet food with eel as a primary ingredient contains higher levels of protein and unsaturated fatty acids compared to those of the pet food made with chicken, especially in terms of omega-3 polyunsaturated fatty acids: the diet containing 14% eel (T1) was 2.44 times that of chicken diet (C), and the diet containing 40% eel (T1) was 2.72 times that of chicken diet (C). Although AAFCO has not defined the minimum requirement of EPA and DHA in adult cat diets (16), previous studies have adequately confirmed the role of omega-3 polyunsaturated fatty acid supplementation in improving cat hair quality and reducing inflammation (21, 22) This study confirmed that continuous feeding with a 14% eel-containing diet for 56 days can significantly increase the hair smoothness of adult cats, while feeding them with a 40% eel-containing diet can improve the hair glossiness and smoothness of cats, which was consistent with previous research findings (23).

Studies have shown that the mechanisms by which omega-3 polyunsaturated fatty acids improve hair include maintaining and further promoting hair growth, reducing inflammation and antioxidants, keeping skin barriers intact, and enhancing absorption and transport of fat-soluble vitamins within the body (22, 24). SEM results of this study showed that after feeding cats with eel-containing food, newly grown hair had lower initial thickness, which is assumed to be the intrinsic for increased glossiness and smoothness of cat hair (15, 25). Unfortunately, the molecular mechanisms behind this process remain unclear.

In terms of antioxidant activity, this study found that after 56 days of consuming cat food with different levels of eel content, cats showed a significant increase in serum total antioxidant capacity. This study conducted by Liu and Zhao (26). Further research is needed to confirm the protective effect on skin barriers and absorption of fat-soluble vitamins.

Obesity has been a major problem for pet owners in recent years. According to Association for Pet Obesity (APOP), the 2022 U. S. Pet Obesity Prevalence Survey found a staggering 61% of cats and 59% of dogs are overweight or have obesity (27). Therefore, effective weight control strategies for pets become paramount. In this study, throughout the entire experimental period, the experimental group consuming the eel-containing diet exhibited a higher average daily food intake compared to the control group, although the difference was not statistically significant (*p* > 0.05). This was a fascinating discovery: in this research, After calculation (28), in this study control diet (chicken): 18.77 MJ/kg, 14% eel-containing diet: 18.35 MJ/kg, 40% eel-containing diet: 17.98 MJ/kg. This indicates that replacing chicken with eel resulted in a slight decrease in dietary metabolizable energy. Meanwhile, studies have shown that supplementation with long-chain polyunsaturated fatty acids can reduce fat tissue deposition in animals (29, 30). In this study, we also observed that cats fed with an eel-supplemented diet exhibited lower serum triglyceride levels. This suggests that a greater proportion of triglycerides may have been metabolized in the eel-supplemented group. It is a pity that this study did not measure the body fat percentage of the cats, precluding confirmation of this hypothesis. Nevertheless, at the very least, reduced serum triglyceride levels are generally beneficial in alleviating certain lipid metabolism-related disorders (31).

This study indicates that eel- containing diet has a higher calcium digestion rate, studies shows it is beneficial for the health of pets, especially elderly pets (32, 33), But the underlying mechanisms of why replacing chicken powder with eels can improve the calcium digestibility in cats remain unclear.

The issue of soft stools is a concern for both pet owners and pet food manufacturers (34), as fecal scoring is often used as a preliminary assessment of the health status of dogs and cats. In this study, there were no significant changes in fecal scores for all groups of cats.

This study indicated that replacing some chicken with eels as the main ingredients in cat food was beneficial for increasing the content of unsaturated fatty acids in cat food, improving the glossiness and smoothness of hair. The mechanism behind this improvement was related to thinner scale structure in hairs (15) and increased total antioxidant capacity of cats. Meanwhile replacing some chicken with eels also enhanced the apparent digestibility rate of calcium without causing rapid weight gain or changes in fecal characteristics in cats. This suggested that replacing chicken with eels as the main ingredients in cat food would not have adverse effects on feline health. In most of the testing indicators, there was no evident advantage demonstrated by comparing 40% eel-containing diet with 14% eel-containing diet. Given that eel itself is more expensive than chicken, it is recommended to add 14% eels contains to cat food.

## 5 Conclusion

Incorporating Japanese eel into cat food can improve hair coat condition and enhance calcium digestibility, as well as increase antioxidant capacity and decrease serum triglyceride of healthy adult cats. This study highlights the potential benefits of using Japanese eel as a valuable ingredient in pet food.

## Data Availability

The original contributions presented in the study are included in the article/Supplementary material, further inquiries can be directed to the corresponding authors.
