# Supplementary material for: The effect of Japanese eel as a main ingredient on hair condition, antioxidant ability, apparent total tract digestibility and body weight gain in cat food
Source: Front Vet Sci. 2025 Jun 16;12:1553320. doi: 10.3389/fvets.2025.1553320 (PMC12206635; doi:10.3389/fvets.2025.1553320)
Supplement: Supplementary file 1 [file Table_1.docx]

Supplementary Material

# Supplementary Material 1: Information of animals

| **Serial number** | **Group** | **variety** | **Cage No.** | **Age** | **gender** | **Group weight  (kg)** |
| --- | --- | --- | --- | --- | --- | --- |
| 1 | C | British Shorthair | 2 | 4 | ♀ | 3.86 |
| 2 |  | Domestic Shorthair | 26 | 2 | ♂ | 2.98 |
| 3 |  | Domestic Shorthair | 18 | 3 | ♂ | 3.26 |
| 4 |  | Domestic Shorthair | 19 | 5 | ♀ | 3.65 |
| 5 |  | British Shorthair | 24 | 3 | ♀ | 4.34 |
| 6 |  | American Shorthair | 4 | 2 | ♂ | 3.35 |
| 7 |  | Ragdoll | 14 | 3 | ♀ | 4.94 |
| 8 |  | Ragdoll | 9 | 3 | ♀ | 2.83 |
| 9 | T1 | British Shorthair | 16 | 3 | ♀ | 4.14 |
| 10 |  | Domestic Shorthair | 27 | 4 | ♀ | 3.08 |
| 11 |  | Domestic Shorthair | 25 | 4 | ♀ | 3.78 |
| 12 |  | Domestic Shorthair | 22 | 4 | ♀ | 3.78 |
| 13 |  | British Shorthair | 28 | 3 | ♂ | 4.05 |
| 14 |  | American Shorthair | 10 | 2 | ♂ | 3.28 |
| 15 |  | Ragdoll | 6 | 3 | ♂ | 4.12 |
| 16 |  | Ragdoll | 12 | 3 | ♀ | 3.65 |
| 17 | T2 | British Shorthair | 7 | 2 | ♀ | 3.56 |
| 18 |  | Domestic Shorthair | 8 | 3 | ♀ | 3.52 |
| 19 |  | Domestic Shorthair | 23 | 5 | ♂ | 3.12 |
| 20 |  | Domestic Shorthair | 21 | 5 | ♀ | 2.28 |
| 21 |  | British Shorthair | 29 | 3 | ♂ | 4.14 |
| 22 |  | American Shorthair | 13 | 2 | ♂ | 3.22 |
| 23 |  | Ragdoll | 15 | 3 | ♀ | 4.39 |
| 24 |  | Ragdoll | 3 | 3 | ♂ | 4.03 |

| Group information statistics | | | | |
| --- | --- | --- | --- | --- |
| Grouping | variety | gender | weight* | |
| C | 2 British Shorthair +3 Domestic Shorthair+1 American Shorthair +2 Ragdoll | 3 males and 5 females | 3.65±0.25 | |
| T1 | 2 British Shorthair +3 Domestic Shorthair+1 American Shorthair +2 Ragdoll | 3 males and 5 females | 3.74±0.14 | |
| T2 | 2 British Shorthair +3 Domestic Shorthair+1 American Shorthair +2 Ragdoll | 4 males and 4 females | 3.53±0.24 | |
| *After testing, there was no statistical difference in body weight among the groups ( *P* > 0.1) | |  |  |  |
